# Research Models and Tools for the Identification of Antivirals and Therapeutics against Zika Virus Infection

**DOI:** 10.3390/v10110593

**Published:** 2018-10-30

**Authors:** Marco P. Alves, Nathalie J. Vielle, Volker Thiel, Stephanie Pfaender

**Affiliations:** 1 Institute of Virology and Immunology, 3012 Bern, Switzerland; nathalie.vielle@vetsuisse.unibe.ch; 2 Department of Infectious Diseases and Pathobiology, Vetsuisse Faculty, University of Bern, 3012 Bern, Switzerland; 3 Graduate School for Cellular and Biomedical Sciences, University of Bern, 3012 Bern, Switzerland

**Keywords:** Zika virus, antivirals, therapeutics, research models and tools

## Abstract

Zika virus recently re-emerged and caused global outbreaks mainly in Central Africa, Southeast Asia, the Pacific Islands and in Central and South America. Even though there is a declining trend, the virus continues to spread throughout different geographical regions of the world. Since its re-emergence in 2015, massive advances have been made regarding our understanding of clinical manifestations, epidemiology, genetic diversity, genomic structure and potential therapeutic intervention strategies. Nevertheless, treatment remains a challenge as there is no licensed effective therapy available. This review focuses on the recent advances regarding research models, as well as available experimental tools that can be used for the identification and characterization of potential antiviral targets and therapeutic intervention strategies.

## 1. Introduction

Zika virus (ZIKV) was first isolated in 1947 in the Zika forest in Uganda from an infected rhesus macaque monkey [1]. Since its discovery, the virus has been detected in different species of *Aedes* mosquitos followed by the identification of the first infected human case in Uganda in 1962 [2,3,4,5]. Since then, human infections had only been sporadically reported in Africa and Asia [6] before the first major outbreak occurred in 2007 in Micronesia [6,7]. After this, the virus continued to spread and caused another major outbreak in 2013–2014 in French Polynesia [8], which was retrospectively associated with an unusual high frequency of newborns with microcephaly, a cerebral congenital anomaly, and an increase in the number of cases of Guillain-Barré syndrome (GBS) in adults [9]. ZIKV received global attention in 2015, following its emergence in Brazil due to its association with several thousand cases of microcephaly in newborn children [10,11,12]. Since then, the virus has spread further, with a total of 86 countries reporting evidence of Zika infection (WHO 2018). Mosquito bites present the major route of transmission of ZIKV, after which viral replication is believed to occur in skin fibroblasts/keratinocytes and skin-associated dendritic cells (DCs) with subsequent dissemination to lymph nodes and the bloodstream [13]. In addition, sporadic non-vector borne human-to-human transmissions have been described. ZIKV has been reported to be transmitted perinatally (reviewed in [14]), sexually [15] and via blood transfusion products [16,17]. Recently, the virus was suspected to transmit through breastfeeding [18]. Furthermore, viral RNA, as well as in some cases infectious virus, has been isolated from various body fluids (reviewed in [14]) including saliva, urine, vaginal secretions, breast milk, semen and conjunctival fluid [19].

ZIKV infection is in most cases asymptomatic or can lead to mild, self-limiting symptoms including rash, fever, joint pain, as well as conjunctivitis [20]. Infrequently, severe neurological conditions such as GBS [21], meningoencephalitis [22] and acute myelitis [23] have been reported in association with ZIKV infection. However, exposure to ZIKV during pregnancy can have severe consequences for the fetus including several fetal malformations such as microcephaly and hydrocephaly, as well as spontaneous abortion, stillbirth and placental insufficiency [20,24]. In addition, it is becoming clear that the long-term neurological defects in affected children are not yet fully elucidated. Up to date, there is still no protective vaccine available. Mosquito control practices are the primary measures to impede further spread and transmission of the virus. Even though much progress was made regarding potential treatment options, no specific antiviral has been licensed against ZIKV, emphasizing the need for the development of effective therapeutics.

## 2. Replication Cycle and Potential Intervention Strategies

### 2.1. Genomic Organization

ZIKV belongs to the genus flavivirus within the Flaviviridae family. It has a positive-sense single-stranded RNA genome of 10.8 kb, containing a single open reading frame, flanked by a 5′ and 3′ untranslated region (UTR), encoding for three structural and seven nonstructural proteins, which are co- and post-translationally processed by viral and host proteases [25]. The three structural proteins, the envelope protein (E), the membrane protein (M), which is expressed in the immature virion as precursor preM, as well as the capsid (C) form the mature virion. The nonstructural (NS) proteins NS1, NS2A, NS2B, NS3, NS4A, NS4B and NS5 are involved in viral replication and assembly and participate in the evasion of the host immune system [26].

### 2.2. Replication Cycle

After binding to specific receptors on the host cell, the virus is internalized by clathrin-mediated endocytosis. The low pH within the endosomes triggers viral uncoating and release of the viral genome into the cytosol [27]. The genomic RNA is translated, resulting in the synthesis of a polyprotein that is co- and post-translationally processed; the RNA-dependent RNA polymerase (RdRp) NS5 replicates viral RNA in close association with cell-derived membranes [28]. Immature virions containing newly synthesized RNA assemble through budding within the endoplasmatic reticulum (ER), followed by transition from the ER through the trans-Golgi network (TGN) to the cell surface, upon which protease- and pH-dependent maturation occurs, and subsequent budding and release of mature virus particles through the secretory pathway [29].

### 2.3. Intervention Strategies

**Figure 1:**
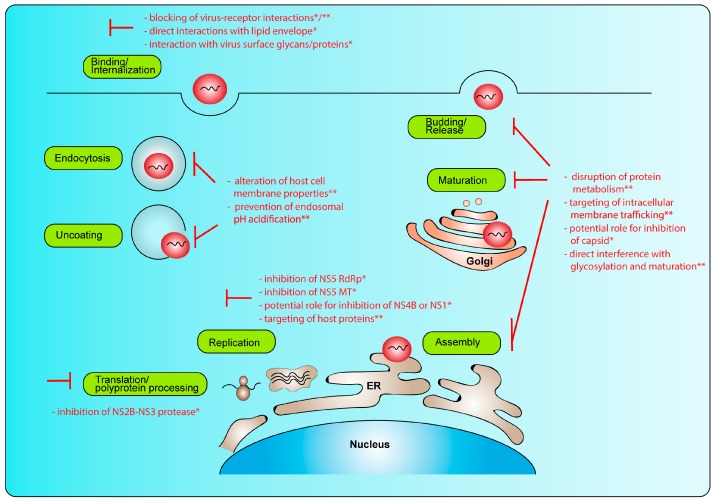
Intervention strategies to interfere with the different stages of the viral replication cycle. (*) direct-acting antivirals; (**) host-targeting antiviral intervention strategies. RdRp: RNA-dependent RNA polymerase, MT: methyltransferase.

Several therapeutic approaches that target the different steps of the viral replication cycle (Figure 1) have been developed (reviewed in [30,31,32]). In general, drug candidates can be classified according to their mode of action, either directed against viral targets (direct-acting antivirals) or acting against cellular targets (host-targeting antivirals). To inhibit the binding of a virus particle to a target cell, strategies include, among others, blocking of the receptor-virus interactions via various compounds [33,34], direct interaction with the viral lipid envelop [35], targeting of the viral surface by structurally mimicking cellular receptors, binding to carbohydrates that interact directly with the glycosylated viral envelope proteins or binding to other peptide regions of the viral envelope (reviewed in [32]). Another antiviral strategy is to target endocytosis and subsequent pH-dependent endosomal fusion and uncoating of the viral particle. This includes, e.g., drugs that alter host-cell membrane properties [36] or blocking of endosomal pH acidification [37,38,39,40,41]. Interference with translation of the viral polyprotein and polyprotein processing via host and viral proteases also represents an attractive target for antiviral drugs. Especially, interference with the viral NS2B-NS3 protease, which post-translationally cleaves the polyprotein, has been extensively evaluated (reviewed in [30,31,32]). Next to its protease activity, NS3 displays a nucleoside-tri-phosphatase (NTPase) and RNA helicase activity that is crucial for viral replication. However, there are only a few studies investigating NS3 helicase inhibitors so far [42]. This might be due to some difficulties, e.g., limited access to the active site of the NS3 helicase-RNA complex as described for Dengue virus (DENV) [43]. NS5 offers a very attractive target for intervention with the viral replication complex, which is one of the most conserved among the NS proteins. NS5 contains the RdRp at the C-terminus, which is required for RNA synthesis, and there are several drugs described to interfere with its function (reviewed in [30,31,32]). Next to its polymerase function, NS5 encodes an N-terminal methyltransferase (MTase) domain, which is required for mRNA capping and stabilization to facilitate the translation process, to escape detection of the viral mRNA by the host innate immune sensors, as well as for cap-independent methylation [44]. Interference with the MTase domain offers an additional therapeutic target [42,45,46,47,48]. Furthermore, interference with other viral proteins that are crucial for viral RNA replication, e.g., NS4B, which has been shown to be a therapeutic target for other flaviviruses [49,50,51,52] or NS1 [53], could be attractive for the development of new antivirals. In addition, host proteins could be targeted to inhibit viral replication. These include host cell nucleoside biosynthesis inhibitors, host cell lipid biosynthesis inhibitors and host kinase inhibitors (reviewed in [53]). To inhibit viral assembly, maturation and budding, interference with the host metabolism by targeting proteins involved in protein synthesis, folding and degradation can be employed (reviewed in [30]). Furthermore, targeting of intracellular membrane trafficking can inhibit several steps of the viral replication cycle, including entry, assembly and release of infectious particles [54]. Compounds that presumably directly interfere with glycosylation and maturation of ZIKV during its trafficking through the secretory pathway can also inhibit the release of infectious particles [55]. Finally, the capsid protein could be targeted by antivirals. The protein has multiple functions during the viral replication cycle, and studies with other flaviviruses have shown an effect of specific capsid inhibitors on viral assembly and/or release and entry of infectious particles [56,57,58]. In conclusion, several strategies have been employed for interfering with the viral replication cycle (Figure 1). However, to avoid the surge of resistance mutation, several strategies could and should be used in combination to treat an acute ZIKV infection.

## 3. In Vitro Models and Screening Approaches

**Figure 2:**
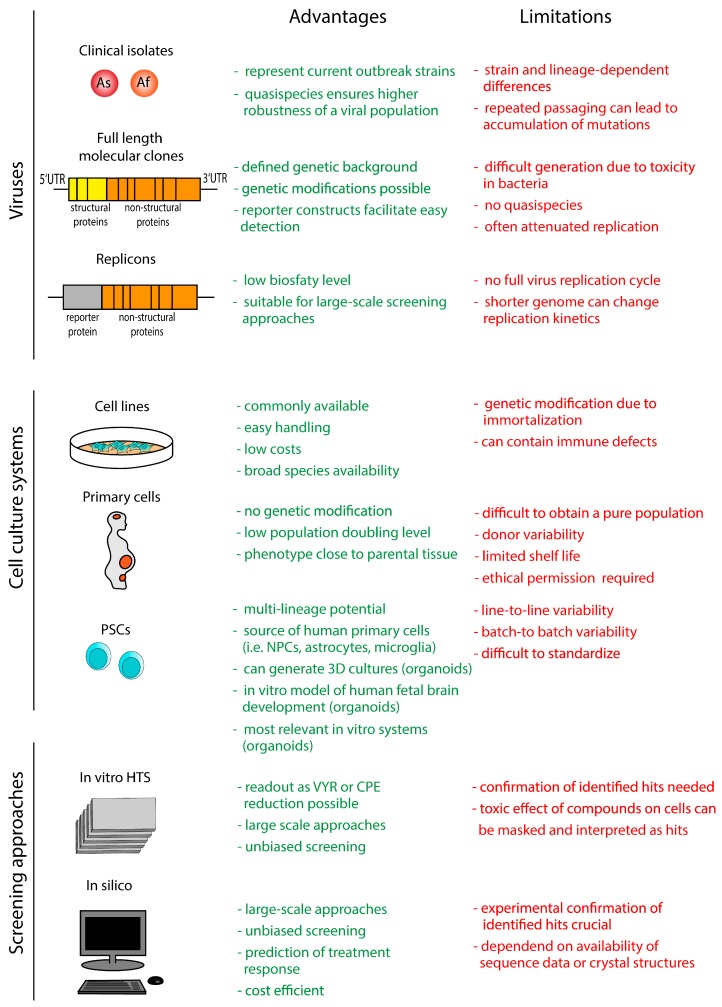
In vitro systems and screening approaches used for antiviral compound development against ZIKV. The text highlighted in green and red indicates advantages and limitations, respectively. PSCs: pluripotent stem cells, HTS: high-throughput screening, VYR: virus yield reduction, CPE: cytopathic effect.

The first prerequisite with respect to the development of antiviral compounds is the availability of in vitro systems including virus isolates and/or recombinant constructs and robust in vitro cellular models (Figure 2).

### 3.1. Viruses

#### 3.1.1. Clinical Viral Isolates

Clinical viral isolates are the best representatives of the currently circulating ZIKV strains. Among ZIKV strains, there are two distinct lineages described, the African and the Asian lineages [59]. Phylogenetic analysis revealed that the currently circulating and pathogenic ZIKV strains most likely descended from the Asian lineage [60]. The “historical” ZIKV strain from Uganda (MR766) has been most consistently used in several in vitro and in vivo studies. Even though it has been passaged over 147 times in different culture models, there seems to be only a low number of nucleotide changes compared to some primary ancient African lineage isolates [61]. Interestingly, of all the ZIKV genomes isolated so far, all African strains have been recovered from mosquitos or non-human primates (NHPs), whereas the Asian strains have been isolated from mosquitos or humans [62]. Phylogenetic analysis revealed that the virus has changed significantly since its initial isolation, with regard to both the nucleotide sequences and amino acid composition with several lineage-specific differences between the African and Asian isolates [59,63,64]. These changes can have a substantial impact on pathogenesis, viral fitness, transmissibility and replication kinetics. Indeed, there are several studies that have analyzed the lineage-dependent properties in animal and cell culture models and found differences between the African and the Asian lineages with respect to plaque phenotype, infectivity, replication kinetics and virulence, with most studies showing that the African lineages display faster replication kinetics, high viral titers, as well as higher virulence in mice [64,65,66,67,68]. On the other hand, there is a significant number of studies with primary human cells and mouse models suggesting that the strains from the African lineage are also neurovirulent and sometimes even more than the contemporary Asian strains [64,69,70,71,72,73]. Thus, there is currently no definitive evidence that the rise in microcephaly cases is exclusively caused by the Asian lineage viruses [74,75]. Of note, all these results have to be interpreted with care as variations including viral strain passage number, cell culture system used and utilization of different mouse background strains can have an impact on the observed phenotypes. For instance, it has been speculated that a high passage number of some ZIKV isolates has resulted in a distinct loss of glycosylation sites, which could have an impact on ZIKV-associated pathogenesis [61]. Nevertheless, for the development and characterization of antivirals, it is important to take into account potential differences between viral isolates, as well as culture systems, since variations in infectivity and replication kinetics can have a significant influence on drug efficacy.

#### 3.1.2. Full-Length Infectious Clones

Next to clinical isolates, reverse genetic systems for the generation of recombinant ZIKV are of great advantage to study virus properties, including virulence/attenuation, replication, host range and tropism. Especially with respect to the identification and development of antivirals, molecular infectious clones are crucial for the understanding of the molecular mechanisms of viral replication and consequently development of potential intervention strategies. To generate an infectious clone, a complementary DNA (cDNA) copy of an RNA virus is usually stably incorporated into a plasmid vector and amplified in *Escherichia coli*. This cDNA can be modified and manipulated as required before genomic RNA is obtained via in vitro transcription and subsequent virus recovery upon transfection of suitable cells [76]. Generation of a ZIKV infectious clone has not been that simple to establish as flavivirus sequences are often not compatible with growth in *E. coli* and may contain viral proteins with bacterial toxicity expressed through cryptic bacterial promoters, which hamper the propagation of the viral cDNA via bacterial plasmids (reviewed in [76,77]). Nevertheless, several strategies have been reported to generate ZIKV molecular clones (Table 1). These include the insertion of a synthetic intron into the molecular construct, which is later removed upon splicing in mammalian cells [78,79], or the separation of the viral genome and generation of subgenomic fragments. Several approaches have been used to circumvent toxicity in bacteria including usage of low copy plasmids [80], usage of bacterial artificial chromosomes [81] and separation of the genome to disrupt toxic regions [82,83]. Furthermore, a more elaborated approach using a bacterium-free reverse genetic method called ISA (infectious subgenomic-amplicons) [84] based on the in vitro generation of overlapping subgenomic amplicons covering the ZIKV genome, subsequent transfection into susceptible host cells and recovery of recombinant virus by “in cellulo” recombination was applied [85,86,87]. Furthermore, a circular polymerase extension cloning (CPEC) protocol [88] based on the polymerase extension mechanism to join overlapping DNA fragments into a double-stranded circular form has been used [89,90]. Another recent approach used mutational inactivation of several cryptic *E. coli* promoters [91]. All these efforts have resulted in the construction of several molecular clones encompassing various viral strains, with or without the presence of reporter proteins for easier detection. These proteins can easily be manipulated to study molecular functions and to identify potential targets for therapeutic intervention (Table 1). Furthermore, the appearance of possible resistance mutations can be studied upon repeated passaging of the virus in the presence of a compound of interest, thereby anticipating possible viral counter mechanisms. Nevertheless, it is important to keep in mind that, due to a high mutation rate of viral RNA polymerases, it is believed that RNA viruses exist not as a homogeneous entity, but rather as quasispecies of genomes that are centered around a consensus sequence, which facilitates a higher robustness of a viral population [92,93], features that are not recapitulated in molecular clones. This leads, for most ZIKV clones described so far, to an attenuation in cell lines in comparison to the parental strains. Furthermore, reporter-protein expression can additionally attenuate viral replication and/or can show low genomic stability limited to few passage numbers.

#### 3.1.3. Replicons

Compared to full-length infectious clones, ZIKV replicons offer an alternative for the identification and characterization of antiviral compounds. Subgenomic replicons contain the genetic elements crucial for viral replication, but lacking the structural genes required for assembly and release of infectious progeny virus [94]. This renders the replicon system non-infectious, thereby reducing possible biosafety concerns, an issue to consider when used for large screening approaches of compound libraries. In general, flavivirus replicons encompass an in-frame deletion in the structural genes; however, the first 20 codons of the C protein, as well as the 3′ end of the E gene need to be retained as they contain important elements for replication, as well as the signal peptide for the downstream NS1 protein, respectively [94]. Incorporation of a reporter protein, either luminescence- or fluorescence-based, allows for easy detection of viral replication. Stable replicon cell lines can be generated by introducing resistance genes and thus facilitate large-scale antiviral screenings [94]. Several approaches have been described to generate and use ZIKV replicons for the characterization of antivirals [95,96]. Future studies using this molecular tool might facilitate the characterization and development of new therapeutic strategies to inhibit ZIKV replication.

### 3.2. Cell Culture Systems

Cell culture models are crucial tools to study the molecular mechanisms of ZIKV life cycle and to develop and identify therapeutics. ZIKV has been shown to display a broad tissue tropism with several cell surface receptors facilitating ZIKV entry. These include receptor tyrosine kinase family members like AXL and Tyro-3, as well as the innate immune receptor DC-SIGN and the receptor TIM-1, also known as hepatitis A virus cellular receptor 1 (HAVcr-1) (reviewed in [97]). However, none of these receptors seems to be unique for ZIKV. Genetic ablations of, e.g., the AXL receptor, which has been discussed as a crucial attachment factor for ZIKV, could not confer resistance to ZIKV infection in vitro and in vivo [98]. This might indicate that ZIKV either concordantly uses several receptors as attachment factors or that receptor usage differs between different cell types and tissues. In accordance with its clinical manifestation, the virus infects several brain cells including cortical neurons, neuronal progenitor cells (NPCs), as well as glial cells, astrocytes and microglia. Furthermore, given its classical route of transmission, skin cells with dermal fibroblasts and epidermal keratinocytes, as well as skin-associated immune cells, including DCs, have also been described to be susceptible. Other susceptible tissues include testis, with Sertoli cells suspected to act as a ZIKV reservoir, and placenta with Hofbauer cells, trophoblasts and placental endothelial cells being susceptible to ZIKV infection (reviewed in [97]). A comprehensive list of the currently identified susceptible human primary cells to ZIKV infection is presented in Table 2.

#### 3.2.1. Human and Animal Cell Lines

Cell lines can be used as a platform to produce high-titer ZIKV stocks, but also to study various aspects of virus replication, including replication kinetics, susceptibility and, to a certain extent, immune response. Especially for the identification and initial characterization of antivirals, they offer a solid basis and have been used to test several approaches. One of the most commonly-used cell lines in ZIKV research is the Vero cell line derived from epithelial kidney cells from African green monkeys. These cells are deficient in type I interferon (IFN) production, making them suitable to yield high virus titers [129]. Other cell lines that have been shown to be susceptible and support ZIKV replication include human kidney, skin, liver, muscle, retina, placental and lung epithelial cell lines, as well as human and murine neuronal cell lines. Interestingly, even though ZIKV was able to replicate efficiently in various cell lines, the amount of intracellular infectious particles, as well as virus release and extent of cytopathic effect varied among the cell lines tested [87,113,130,131,132]. Furthermore, it was shown that next to human, NHPs and murine cells, ZIKV is able to replicate within a wide range of animal cell lines, expanding the variety of cell lines that can be used to study ZIKV infections [81,130,133]. Given their usefulness regarding availability, cost and handling, cell lines offer a powerful tool for the identification and characterization of antivirals. However, cell line-specific differences with respect to viral kinetics, observed phenotypes, genetic manipulation, as well as innate immune defects should be considered when interpreting results.

#### 3.2.2. Human Primary Cell Cultures

In contrast to cell lines which often differ genetically and phenotypically from their parental tissue, primary cells have a low population doubling level and therefore more closely recapitulate the physiological conditions observed in vivo (Table 2). Primary cells are characterized by their high degree of specialization, are often fully differentiated and thus require optimal defined culture conditions in order to preserve their original phenotype. This means that the use of serum-free specific formulated media is required in order to prevent undesired differentiation or promote the growth of contaminating cells, typically fibroblasts. A large majority of the studies for searching/screening potential therapeutics against ZIKV infection focused on a specific in vitro system often based on cell lines. Considering the wide tissue and cellular tropism of ZIKV and the different possible routes of transmission, broader approaches are mandatory. Thus, when evaluating a potential antiviral approach against ZIKV infection, the selection of the appropriate in vitro system is of central importance, particularly when evaluating compounds targeting cellular host factors involved in viral life cycle. For instance, when the experimental paradigm is to pharmacologically modulate host factors to interfere with vector-borne transmission, the primary cellular targets of ZIKV infection should be selected for therapeutics screening, including skin fibroblasts, epidermal keratinocytes or skin-associated immune cells such as DCs. On the other hand, when screening for therapeutics in the context of vertical transmission, placental, amniotic and/or fetal primary cultures are a more appropriate in vitro system and have already been used by some investigators for the screening of antivirals [34,116,134]. Furthermore, the ability of ZIKV to be transmitted sexually and to persist in the genital tract led to the use of human primary vaginal/cervical epithelial and male germ cells to test the efficacy of anti-ZIKV compounds [121,127]. Although primary cell cultures are relevant biological systems for the identification of potential anti-ZIKV therapeutics, there are technical difficulties limiting their use. Depending on the tissue of interest, obtaining a pure population of primary cells can be laborious, and phenotypic variability is commonly observed between donors. Furthermore, the vast majority of isolated primary cells have a limited shelf life and eventually undergo cellular senescence after a short number of passages. Finally, yet importantly, work with human-derived primary cells from native tissue requires patient-approved consent and the evaluation by an institutional ethical review board.

#### 3.2.3. Human Pluripotent Stem Cell-Derived Cultures

When screening for anti-ZIKV therapeutics targeting host factors with a protective effect on the human neuronal tissue, the use of primary human cell-based systems is challenging for ethical and technical reasons. In addition, in the context of congenital neurotropic ZIKV infection, there is currently no robust in vivo system modelling early human brain development. These obstacles can be bypassed using elaborated in vitro systems exploiting the possibilities offered by human embryonic stem cells (ESCs) and/or human induced pluripotent stem cells (iPSCs) (collectively PSCs) (Table 2). PSCs proliferate extensively and retain multi-lineage activity allowing one to generate virtually any cell type of the body, making them a precious source of primary cells. While ESCs are isolated from surplus human embryos for in vitro fertilization, iPSCs are obtained by reprogramming somatic cells and can be used as a replacement of ESCs to generate distinct brain cells, including cortical neurons, NPCs, glial cells and complex cerebral three-dimensional (3D) cultures termed organoids [135,136]. In the fetal brain, ZIKV targets NPCs, and several investigators used PSC-derived NPCs to investigate the efficacy of antivirals and their ability to prevent microcephaly in vitro [137,138]. Furthermore, there are now established methods to generate primary glial cultures, astrocytes and microglia from PSCs ([139,140,141]. Additionally, a more advanced in vitro system generated from PSCs, namely 3D cerebral organoids, is to date the only available in vitro system modelling early human brain development [142]. Contrary to NPCs and glial cultures, the cerebral organoid approach allows the screening for therapeutics using an in vitro system mimicking the cellular and architectural complexity of the brain tissue and is thus more predictive of how a potential treatment responds in vivo. This advanced in vitro model helped to unravel the link between microcephaly and ZIKV infection during pregnancy [143,144]. Given the neurotropism of ZIKV, brain organoids have already been used to validate anti-ZIKV compounds [38,138,145]. Although the cerebral organoid technology is currently the most relevant model of the developing human brain, this system has some limitations such as PSCs’ line-to-line and organoid batch-to-batch variability, leading to some level of variability and in general to a limited degree of standardization.

### 3.3. Screening Approaches

In addition to testing specific compounds that have been described to inhibit related viruses or have been shown to possess a known antiviral activity, unbiased screening approaches offer an alternative to identify new antiviral compounds based on libraries containing hundreds of bioactive molecules. Several of these libraries contain FDA-approved drugs and clinical investigational agents, which have shown good safety and pharmacokinetics in vivo, thereby facilitating drug-repurposing strategies. Drug repurposing plays a very important role, given that a considerable risk of an infection exists during pregnancy, which can complicate medical intervention. Identification of antiviral drugs that are already approved during pregnancy could greatly accelerate the availability of therapeutic interventions.

#### 3.3.1. In Vitro Screening Approaches

Different in vitro screening approaches exist, which are usually based on an automated readout, mostly microscopy- or luminescence-based. This readout can be either based on virus yield reduction (VYR) or on cytopathic effect (CPE) reduction [30]. VYR assays include luciferase reporter activity assays, where the reduction in reporter signal correlates to VYR, which in the case of ZIKV has been described either upon usage of replicon cell lines [95,96], pseudo-viruses [146], full-length infectious clones [80,83,91], assays based on immuno-labeling of viral proteins and/or cell proliferation markers and fluorescent or histochemical microscopic analysis [34,134,138,147] and assays based on quantification of viral RNA via quantitative PCR [148]. CPE reduction assays generally monitor the ability of the compound to reduce or inhibit virus-mediated CPE, thereby monitoring cell viability [30]. In the case of ZIKV, several assays have been utilized including colorimetric or luminometric cell death assays [149,150,151], bright-field microscopy-based phenotypic assays analyzing the percentage of cells undergoing ZIKV-induced morphology changes detected by automated image analysis software [152], viral plaque forming assays [153] and assays measuring caspase-3 activity, which precedes cell death in ZIKV-infected human NPCs [38]. However, CPE reduction assays lack some specificity as alterations in the cellular morphology or viability based on the compound treatment can sometimes not be distinguished from the effects mediated by a reduced CPE due to reduced virus replication and should therefore be interpreted carefully. An alternative approach is to utilize purified recombinant ZIKV proteins, e.g., the NS2-NS3 protease or the NS5 RdRp, and to monitor their enzymatic activity upon compound treatment [154,155] or utilization of a split luciferase complementation assay for the identification of orthosteric inhibitors, which block specific viral protein interactions (e.g., NS2B-NS3) [156].

#### 3.3.2. In Silico Screening Approaches

Computational in silico approaches have proven to be powerful tools in predicting drug targets and the evaluation of treatment strategies. In general, these in silico systems use computational models to describe macromolecule–ligand interactions. Structure-based approaches mimic the binding of the putatively active ligand within the relevant binding site and are therefore frequently able to facilitate further chemical optimization of the compound [157]. Structure-based drug discovery approaches include in silico homology modeling, which predicts the tertiary structure of an amino acid sequence based on a homologous experimentally-determined structure [158]. Homology modeling techniques and molecular docking analyses, which essentially reflect the ligand-receptor binding process of a virtual library of compounds, can be utilized to identify compounds that can target directly viral proteins and have been applied to ZIKV protease [42,159], methyltransferase [42] and RdRp [42,160]. Furthermore, homology models have been designed to predict the ability of potential compounds to inhibit allosterically the binding of viral envelope proteins to cellular glycoproteins [161]. As an alternative to homology modeling, consensus scoring can be used if the target 3D structure is known. This method involves the comparison of two or more methods of docking process, thereby enhancing the performance of virtual screening by improving the rates at which potential hit compounds are reached, which improves the prediction of bound conformations and poses [162]. As ZIKV proteins have been crystalized and ZIKV virions have been studied by electron microscopy [163,164,165], this method could be used to identify potential hit compounds that have good binding affinities for a real matured ZIKV protein structure obtained with cryo-electron microscopy [166]. A complementary approach to that of molecular docking analysis is to use pharmacophore searching algorithms to find ligands similar to one of known activity and chemical features (such as hydrogen bonds, charges, lipophilic areas) [167,168], which has recently been utilized to identify potent inhibitors of ZIKV NS2B-NS3 [169]. Next to structure-based drug discovery approaches, mathematical analysis of viral replication dynamics and antiviral treatment strategies have been performed for several viruses, including human immunodeficiency virus, hepatitis C virus, influenza A virus, Ebola virus, DENV and ZIKV [170]. A recent study predicted ZIKV replication dynamics in an NHP model including the potential effects of antiviral treatments, data that can be used to optimize and evaluate in silico the impact of antiviral strategies [171]. In conclusion, in silico screening approaches accelerate the drug development process combined with low costs.

## 4. In Vivo Models

**Figure 3:**
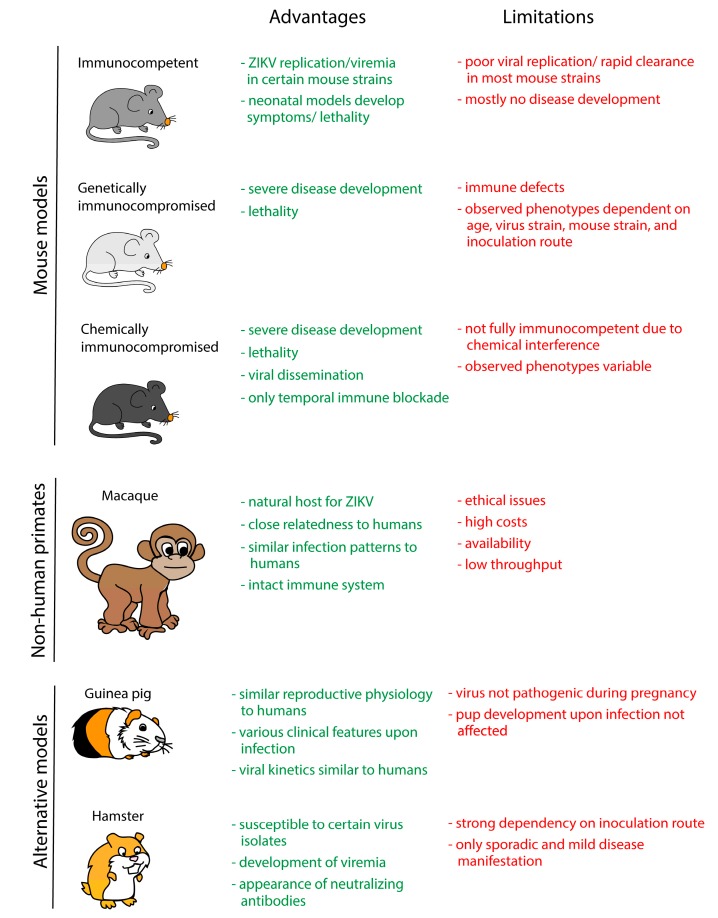
Overview of the currently available in vivo models for the study of ZIKV pathogenesis and for possible antiviral approaches. The text highlighted in green and red indicates advantages and limitations, respectively.

In vitro models greatly facilitate the initial screening and identification of new antivirals. However, they do not fully reflect the complexity and physiology of living organisms. To this end, several in vivo models have been developed to study virus pathogenesis and to test new drugs and vaccines (Figure 3).

### 4.1. Mouse Models

Mouse models are widely used to study various aspects of virus infection in vivo, such as pathogenesis and transmission. Several mouse models have been developed to study ZIKV infection (Table 3). Very early studies have shown that mice are susceptible to ZIKV infection, with intracranial inoculation causing neurological symptoms in suckling mice, whereas consistent disease in adult white mice was not clearly manifested ([172,173,174]. It has been shown that peripheral inoculation of most wildtype (WT) mice results in poor replication, as well as a rapid clearance of infection with no disease development [70,175,176]. One major obstacle appears to be innate immune responses, namely the murine IFN system [177,178]. Similarly to other WT mouse strains, ZIKV infection of the Balb/c mouse model does not result in a fatal outcome; however, it seems to support ZIKV replication more efficiently compared to C57BL/6 WT mice and leads to viremia that is comparable to human in its magnitude and duration [179]. Furthermore, neonatal mouse models have been shown to be susceptible to ZIKV and to develop symptoms and even lead to fetal death depending on the age and virus dose [180,181,182,183]. Another exception seems to be the WT SJL mouse model, which supports ZIKV replication and results after intravenous ZIKV inoculation of pregnant mice in growth restriction of developing fetuses, cortical malformations and ocular defects [144,179]. However, SJL mice have been described to have impaired IFN signaling and several immunological defects, rendering them not fully immunocompetent, which might explain the observed susceptibility and fetal pathogenesis [178,184,185]. To overcome these limitations, immunocompromised mouse systems have been developed. Especially, interference with the type I IFN signaling pathway has been proven to be useful to facilitate viral replication [186]. Genetically modified mice lacking either the type I IFN receptor ifnar1 gene (Ifnar1^−/−^) or the transcription factors Irf3, Irf5 and Irf7 (Irf3^−/−^, Irf5^−/−^, Irf7^−/−^ triple knockout) in 129 (A129) or C57BL/6 background have been shown to be highly susceptible to ZIKV infection and to develop severe diseases following peripheral inoculation, including development of neurological disease and death [70,175,176,180,187,188,189,190]. Severity of ZIKV infection in these immunocompromised mice seems to be age dependent, with older mice (>11 weeks-old) being less susceptible to infection than younger mice (3–5 weeks-old) [175,176]. Double knockout mice, lacking both the type I IFN receptor and type II IFN receptor (AG129), displayed even greater susceptibility and more severe disease following ZIKV infection compared to Ifnar1^−/−^-deficient A129 mice [175,191]. Furthermore, in an attempt to better model vertical ZIKV transmission, it could be shown that crossing of female immunocompromised mice with WT males produced heterozygous, IFN competent (Ifnar^+/−^) fetuses, which better resembled the immune status of human fetuses, with ZIKV maternal inoculation resulting in fetal demise that was associated with ZIKV infection of the placenta and fetal brain [192]. Besides directly targeting the IFN receptors, blockage of type I and type III IFN signaling via interference with STAT signaling in Stat1^−/−^- or Stat2^−/−^-deficient mice has also been shown to facilitate ZIKV infection and spreading [70,193]. Furthermore, blockage of type I IFN signaling upon administration of a monoclonal antibody (Mab) targeting the Ifnar1 receptor has been tested as a means to only temporally block IFN signaling in an otherwise immunocompetent mouse and has been shown to increase susceptibility to ZIKV infections [176,194,195,196]. Increased susceptibility to ZIKV infection has also been demonstrated by pretreatment of mice with dexamethasone, resulting in temporal immunocompromised animals, rendering them susceptible to ZIKV infections [197]. Recently, a transgenic immunocompetent mouse model has been described in which murine Stat2 was replaced with human STAT2, thereby allowing evasion of IFN signaling cascades in infected cells. This model was combined with the generation of a mouse-adapted strain of ZIKV containing a key mutation in the viral NS4B gene and resulted in reduced induction of IFN-β and greater replication in the brain. This approach of establishing an immunocompetent mouse model of ZIKV infection allowed analyses on virus replication, trans-placental transmission, as well as fetal infection [196]. Mouse models can be very well suited to analyze pharmacokinetics of drug candidates, including stability and in vivo retention, as well as the protective efficacy of antivirals and new therapeutics in vivo. There are several studies showing that infection of immunocompromised AG129 (IFN type I and II receptors deficiency), A129 (IFN type I receptor deficiency) or Stat1^−/−^ mice with ZIKV and subsequent treatment with potential antiviral compounds can lead to decreased viral burden in various tissues, reduced pathology, as well as reduced or delayed morbidity and mortality [156,193,198,199,200,201,202,203]. Furthermore, dexamethasone-immunosuppressed mice have been used to evaluate the therapeutic potential of antivirals [159], and in addition, immunocompetent or neonatal mouse models have been shown to be useful to evaluate the efficacy of potential antiviral candidates to reduce viremia [40,204,205,206,207]. Even though mouse models can give important insights into viral transmission and pathogenesis and can be used for the initial characterization of therapeutics, it is important to keep in mind that they differ significantly from humans, with respect to several features including placental anatomy and gestation period, as well as disease manifestation, rendering more complex animal models necessary to study specific aspects of ZIKV infection.

### 4.2. Non-Human Primate Models

NHPs seem to be natural hosts for ZIKV and are believed to represent the major animal reservoir [209]. ZIKV was first isolated from a rhesus macaque [2], and several monkey species in forests were found to be seropositive for ZIKV [210]. Given their close relatedness to humans, they offer great potential as surrogate models to study specific aspects of ZIKV infection and pathogenesis. It has been shown that macaques are susceptible to infection by both African and Asian ZIKV lineages and that infection results in similar patterns comparable to human infections regarding transmission, cellular tropism, immune responses and viral replication. Infection with ZIKV resulted in viremia that peaked around 2–6 days post-infection, followed by viral clearance. Importantly, viral RNA could be detected in various body fluids including urine, saliva, seminal fluid, vaginal secretions and cerebrospinal fluid, indicating similar dissemination patterns compared to humans [211,212,213,214,215,216,217]. Furthermore, mucosal viral inoculation (vaginal or rectal) was shown to lead to productive infection in macaques, mimicking the anogenital transmission route in humans [218]. Importantly, in contrast to mice, NHPs display similarities in gestation, placental barrier and fetal development to humans, rendering them suitable to model ZIKV infections physiologically during pregnancy in humans [219,220]. Indeed, subcutaneous ZIKV inoculation of pregnant macaques with an Asian lineage ZIKV resulted in vertical maternal-fetal ZIKV transmission and reduced growth of the fetal brain, as well as substantial pathology to the central nervous system [216,221]. Even though NHPs offer a more physiological animal model for the study of ZIKV, there are several limitations including ethical issues, high costs, availability and low throughput. These limitations have so far hampered the usage of NHPs for the development and characterization of vaccines and antiviral approaches [214,222,223,224,225].

### 4.3. Alternative Animal Models

Several other animal models have been investigated for the study of ZIKV infections, including guinea pigs, cotton-rats and rabbits, all of which failed to show clinical signs of infection after intracerebral inoculation with the prototype African ZIKV strain MR766 [172]. However, recent studies described the susceptibility of guinea pigs to infection with Asian strains of ZIKV and the development of various clinical features, immune activation and viral kinetics, similar to what has been observed in humans [226]. In addition to subcutaneous infection, other infection routes including intranasal infection and contact transmission of Zika virus could be demonstrated using the guinea pig model, highlighting its suitability to study transmission pathways [227]. However, not all clinical aspects can be reproduced, as circulating ZIKV strains seemed not to be pathogenic during the pregnancy of immunocompetent guinea pigs and did not interfere with offspring development [228]. Nevertheless, in contrast to other small animal models, the guinea pig model is physiologically and immunologically more similar to humans [229]. Especially in the context of ZIKV infection, the similarity of the guinea pig’s reproductive physiology, placentation and estrous cycle to humans, as well as the fact that pups are born with a mature central nervous system can be of great advantage for the study of ZIKV pathogenesis and transmission [229,230,231]. Next to guinea pigs, Syrian golden hamsters have recently also been shown to be to some extent susceptible to certain ZIKV isolates and dependent on the route of inoculation, development of viremia and appearance of virus neutralizing antibodies, and in some cases, mild disease manifestations were observed, rendering them an interesting model to study phenotypic variations between ZIKV strains [232].

These studies show that the host range of ZIKV might be broader than originally anticipated, and different animal models might be useful for the study of different aspects of ZIKV infections. However, the value of these alternative animal models for the characterization and development of antivirals and therapeutics still needs to be fully explored.

## 5. Concluding Remarks

In recent years, significant advances were made regarding our understanding of ZIKV-associated morbidity in human. Consequently, significant progress was achieved regarding the availability of research models and new experimental tools for the identification of therapeutic and antiviral strategies against ZIKV. However, treatment remains a challenge. Given that treatment needs to be safe and efficient especially in the context of pregnancy further complicates drug development. In addition, a declining trend in ZIKV infections could limit clinical trials. As there is no licensed effective therapy available yet, further efforts are required in order to find potential drug candidates with ideally a wide spectrum of action against flaviviruses found in similar geographical regions with often overlapping symptoms. Furthermore, assuming the broad cellular tropism and the different routes of ZIKV transmission of ZIKV, a variety of models should be applied in order to validate a promising drug. Ultimately, beyond the potential clinical benefits, the knowledge derived from the screening of anti-ZIKV drugs using relevant models will reveal new insights into the understanding of the molecular mechanisms of ZIKV-associated morbidity and greatly contribute to increase our knowledge on flavivirus biology.

## Figures and Tables

**Table 1:** Reverse genetic systems for ZIKV, full-length infectious clones.

ZIKV Isolate	Lineage	Isolation	Cloning Strategy	Plasmid Name	Reporter	Tested	Reference
Paraiba_01/2015	Asian	Brazil, 2015	Synthetic intron insertion	ZIKV-*1*	-	Cell culture	[78]
				ZIKV-*ICD*			
				ZIKV-*NS3m*			
MR766	African	Uganda, 1947	Synthetic intron insertion		-	Cell culture	[79]
FSS13025	Asian	Cambodia, 2010	Subgenomic fragments	pFLZIKV	RLuc	Cell culture mosquito-mouse model	[80]
MR766	African	Uganda, 1947	Subgenomic fragments		-	Cell culture mouse model	[82]
H/PF/2013	Asian	French Polynesia, 2013					
SPH2015	Asian	Brazil, 2015					
BeH819015	Asian	Brazil, 2015					
BeH819015	Asian	Brazil, 2015	Subgenomic fragments	icDNA BeH819015	nLuc	Cell culture	[83]
					GFP		
					mCherry		
MR766^NIID^	African	Uganda 1947	Subgenomic fragments	ZIKV-MR766^NIID-MC^	GFP	Cell culture	[85]
				ZIKV_GFP_			
PF	Asian	French Polynesia, 2013	Subgenomic fragments	PF	-	Cell culture	[86]
DAK	African	Dakar, 1984		DAK			
(MART)	Asian	Martinique, 2015		PF/DAK			
				DAK/PF			
				PF/MART			
MR766^M^	African	Uganda, 1947	Subgenomic fragments	MR766^MC^	-	Cell culture	[87]
BeH819015	Asian	Brazil, 2015		BR15^MC^			
				CHIM			
ZIKV_Natal_	Asian	Natal, 2015	Subgenomic fragments			Cell culture	[89]
PRVABC59	Asian	Puerto Rico, 2015	Subgenomic fragments			Cell culture mosquito-mouse model	[90]
MR-766	African	Uganda, 1947	Subgenomic fragments	pBac/MR-766,	-	Cell culture	[81]
P6-740	Asian	Malaysia, 1966		pBac/P6-740			
PRVABC-59	Asian	Puerto Rico, 2015		pBac/PRVABC-59			
MR766	African	Uganda 1947	Mutational inactivation CEPs	synZIKV-MR766	*RLuc*	Cell culture	[91]
H/PF/2013	Asian	French Polynesia, 2013		synZIKV-H/PF/2013	FP635		

nLuc, nano-luciferase; RLuc, Renilla luciferase; FP635, turbo far-red fluorescent protein; icDNA, infectious cDNA clone; CEPs, cryptic *E. coli* promoters.

**Table 2:** Susceptible human primary cells to ZIKV infection.

Primary Cell Type	Tissue/Source	Reference
Dermal fibroblasts	Skin	[99,100]
Epidermal keratinocytes	Skin	[99]
Blood dendritic cells	Peripheral blood	[101]
Monocyte-derived dendritic cells	Peripheral blood	[73,99,102,103]
Monocyte-derived macrophages	Peripheral blood	[104,105]
Monocytes	Peripheral blood	[104,106,107]
NPCs	Brain/PSCs	[108,109]
Astrocytes	Brain	[66,110,111,112]
Microglia	Brain	[110]
Endothelial cells	Brain	[113]
Hofbauer cells	Placenta	[114,115,116,117]
Trophoblasts	Placenta	[114,116,118,119,120]
Fibroblasts	Placenta	[116]
Endothelial cells	Placenta	[116]
Fibroblasts	Uterus	[34,114]
Mesenchymal stem cells	Umbilical cord	[114]
Epithelial cells	Vagina and cervix	[121]
Sertoli cells	Testis	[122,123,124]
Spermatozoa	Testis	[125]
Germ cells	Testis	[126,127]
Retinal endothelial cells	Eye	[128]
Retinal pericytes	Eye	[128]
Retinal pigmented cells	Eye	[128]

**Table 3:** Mouse models of ZIKV infection.

Model	Strain	Deficiency	ZIKV Inoculation	Pathogenesis	Reference
Immunocompetent	Balb/c		ZIKV2015 (Brazil, 2015)	ZIKV replication	[179]
			PRVABC59 (Puerto Rico, 2015)	Viremia	
				Lethality: No	
	SJL	Immunological defects	ZIKV2015 (Brazil, 2015)	ZIKV replication	[179]
				Viremia	
				Lethality: No	
			ZIKV^BR^ (Brazil, 2015)	Whole-body growth delay or intra-uterine growth restriction (IUGR) in pups	[144]
Neonatal	C57BL/6		PRVABC59 (Puerto Rico, 2015)	Neurological symptoms	[180]
	C57BL/6		MR766 (Uganda, 1947)	Neurological symptoms	[183]
	Kunming		PRVABC59 (Puerto Rico, 2015)	Lethality: Yes (age and dose-dependent)	
	ICR		SZ-WIV01 (China, 2016)		
	Balb/c		Z16006 (China, 2016)	Neurological symptoms	[181]
	C57BL/6			Lethality: Yes/No (mouse strain-specific differences)	
	Kunming				
	Balb/c		MRS_OPY_Martinique_PaRi_2015	Neurological symptoms	[182]
			(Martinique, 2015)	Lethality: Yes (viral strain-specific differences)	
			GZ01 (Venezuela, 2016)		
			SZ01 (Samoa, 2016)		
			FSS13025 (Cambodia, 2010)		
	C57Bl/6		Dakar 41519 (Senegal, 1984)	Lethality: Yes (partly)	[176]
Partially immunocompetent	hSTAT2 KI Mice	hSTAT2 under control mStat2 promotor	Mouse adapted ZIKV-Dak-41525 (=ZIKV-Dak-MA) (Senegal, 1984)	Placental transmission	[196]
	(C57BL/6)			Lethality: Yes (partially)	
Genetically Immunocompromised	A192 (129sV)	Ifnar1^−/−^	MP1751 (Uganda, 1962)	Sever disease	[187]
				Lethality: Yes	
			FSS13025 (Cambodia, 2010)	Signs of illness	[175]
				Severe disease (age dependent)	
				Lethality: Yes (age dependent)	
	IFNAR^−/−^	Ifnar^−/−^	MR 766 (Uganda, 1947)	Severe disease	[176]
	(C57BL/6)		Dakar 41519, 41667, 4167 (Senegal, 1984)	Neurological symptoms	
			H/PF/2013 (FP, 2013)	Lethality: Yes (age dependent)	
			PRVABC59 (Puerto Rico, 2015)	Severe disease	[180]
				Lethality: Yes	
			FSS13025 (Cambodia, 2010)	Severe disease	[188]
				Lethality: Yes	
			MR 766 (Uganda, 1947	Severe disease	[70]
			DAKAR 41519 (Senegal, 1984)	Neurological symptoms	
			P6-740 (Malaysia, 1966)	Lethality: Yes	
			FSS13025 (Cambodia, 2010)	(virus strain-specific differences in morbidity and lethality)	
			PRVABC59 (Puerto Rico, 2015)		
	Irf3^−/−^, Irf5^−/−^, Irf7^−/−^ TKO	Irf3^−/−^, Irf5^−/−^, Irf7^−/−^	MR 766 (Uganda, 1947),	Severe Disease	[176]
	(C57BL/6)		H/PF/2013 (French Polynesia, 2013)	Neurological symptoms	
				Lethality: Yes	
			FSS13025 (Cambodia, 2010)	Signs of disease	[189]
				Neurological symptoms	
	Irf3^−/−^, Irf7^−/−^ DKO	Irf3^−/−^, Irf7^−/−^	MR766 (Uganda, 1947)	Viremia	[190]
	(C57BL/6)			Lethality: Infrequent	
	AG129 (129/Sv)	Ifnar^−/−^, Ifngr1^−/−^	FSS13025 (Cambodia, 2010)	Severe disease	[175]
				Neurological symptoms	
				Lethality: Yes	
			H/PF/2013 (French Polynesia, 2013)	Severe disease	[191]
				Lethality: Yes	
	Stat2^−/−^ (C57BL/6)	Stat2^−/−^	MR 766 (Uganda, 1947)	Severe disease	[70]
			DAKAR 41519 (Senegal, 1984)	Neurological symptoms	
			P6-740 (Malaysia, 1966)	Lethality: Yes	
			FSS13025 (Cambodia, 2010)	(virus strain-specific differences in morbidity and lethality)	
			PRVABC59 (Puerto Rico, 2015)		
	Stat1^−/−^	Stat1^−/−^	MR 766 (Uganda, 1947)	Viremia	[193]
				Disease development	
				Lethality: Yes	
Chemically immunocompromised	C57Bl/6	IFNAR1-blocking monoclonal antibody (MAb-5A3	H/PF/2013 (French Polynesia, 2013)	Viremia/ Increased replication	[176]
				Lethality: No	
			DAK AR D 41525 (Senegal, 1984)	Viremia	[195]
				Severe disease	
				Lethality: Yes (differences depending on inoculation route)	
			Mouse adapted ZIKV-Dak-41525 (=ZIKV-Dak-MA) (Senegal, 1984)	Viremia	[194]
				Severe Disease	
				Lethality: Yes	
			ZIKV-DAK-41525 (Senegal, 1984)	Lethality: Yes	[196]
			ZIKV-DAK-MA (mouse adapted)		
	C57BL/6		H/PF/2013 (French Polynesia, 2013)	Viremia in testis and epididymis	[208]
			Mouse adapted ZIKV-Dak-41525 (=ZIKV-Dak-MA) (Senegal, 1984)		
	Balb/c	Dexamethasone	PRVABC59 (Puerto Rico, 2015)	Viral dissemination	[197]
				Severe disease (after withdrawal)	
				Lethality: Yes (after withdrawal)	
